# Hematopoietic Cells Derived from Cancer Stem Cells Generated from Mouse Induced Pluripotent Stem Cells

**DOI:** 10.3390/cancers12010082

**Published:** 2019-12-29

**Authors:** Ghmkin Hassan, Said M. Afify, Neha Nair, Kazuki Kumon, Amira Osman, Juan Du, Hager Mansour, Hagar A Abu Quora, Hend M Nawara, Ayano Satoh, Maram H. Zahra, Nobuhiro Okada, Akimasa Seno, Masaharu Seno

**Affiliations:** 1 Graduate School of Interdisciplinary Science and Engineering in Health Systems, Okayama University, Okayama 700-8530, Japan; saidafify@s.okayama-u.ac.jp (S.M.A.); pxe25xvd@okayama-u.ac.jp (A.O.); pfgg90pn@s.okayama-u.ac.jp (H.A.A.Q.); ayano113@cc.okayama-u.ac.jp (A.S.); maram@okayama-u.ac.jp (M.H.Z.); okadan@okayama-u.ac.jp (N.O.); aseno@okayama-u.ac.jp (A.S.); 2 Department of Microbiology and Biochemistry, Faculty of Pharmacy, Damascus University, Damascus 10769, Syria; 3 Division of Biochemistry, Chemistry Department, Faculty of Science, Menoufia University, Shebin El Koum-Menoufia 32511, Egypt; 4 Graduate School of Natural Science and Technology, Okayama University, Okayama 700-8530, Japan; nehand2013@gmail.com (N.N.); en419781@s.okayama-u.ac.jp (K.K.); djmail@yeah.net (J.D.); pcnp49mj@s.okayama-u.ac.jp (H.M.); pnt32hvi@s.okayama-u.ac.jp (H.M.N.); 5 Department of Histology, Faculty of Medicine, Kafrelsheikh University, Kafr Elsheikh 32511, Egypt; 6 Integrative Biosciences Center, Okayama University Research Laboratory for Stem Cell Engineering in Detroit, Wayne State University, Detroit, MI 48202, USA

**Keywords:** Induced pluripotent stem cells, Cancer stem cells differentiation, tumor microenvironment, hematopoietic cells

## Abstract

Cancer stem cells (CSCs) represent the subpopulation of cancer cells with the ability to differentiate into other cell phenotypes and initiated tumorigenesis. Previously, we reported generating CSCs from mouse induced pluripotent stem cells (miPSCs). Here, we investigated the ability of the CSCs to differentiate into hematopoietic cells. First, the primary cells were isolated from malignant tumors that were formed by the CSCs. Non-adherent cells (NACs) that arose from adherent cells were collected and their viability, as well as the morphology and expression of hematopoietic cell markers, were analyzed. Moreover, NACs were injected into the tail vein of busulfan conditioned Balb/c nude mice. Finally, CSCs were induced to differentiate to macrophages while using IL3 and SCF. The round nucleated NACs were found to be viable, positive for hematopoietic lineage markers and CD34, and expressed hematopoietic markers, just like homing to the bone marrow. When NACs were injected into mice, Wright–Giemsa staining showed that the number of white blood cells got higher than those in the control mice after four weeks. CSCs also showed the ability to differentiate toward macrophages. CSCs were demonstrated to have the potential to provide progenies with hematopoietic markers, morphology, and homing ability to the bone marrow, which could give new insight into the tumor microenvironment according to the plasticity of CSCs.

## 1. Introduction

Cancers have complex architecture, in which the malignant cells interact with non-transformed cells, forming the tumor microenvironment (TME) [1]. TME is considered to consist of different types of cells, including fibroblasts, endothelial cells, adipocytes, and immune cells, such as macrophages, myeloid-derived suppressor cells, and lymphocytes. TME often promotes tumor development in different stages of carcinogenesis. The identification of different components of TME, to which the tumor cells respond, could help to understand the chemoresistance in more detail and contribute to the development of more effective therapy [2,3].

Blood vessels and immune cells are considered as the main components of the TME influencing the progression and growth of tumors. Different types of leukocytes are known to be present in many different types of cancers [4,5]. Macrophages are one of the important components of the TME. Tumor-associated macrophages (TAMs) are located in the tumor, where they support the growth and progression of tumors by responding and secreting many interleukins and chemokines [6,7]. Recent studies have revealed that TAMs are not specifically derived from circulating monocytes, but from embryonic macrophages. Moreover, the origins of TAMs are currently a matter of debate and area for future research. In this context, leukocytes can interact with cancer cells that are interfering with tumor progression or promoting tumor growth [8].

On the other hand, cancer stem cells (CSCs) are thought to be responsible for initiating tumors with the ability to self-renew and differentiation potential. CSCs were detected and isolated from various cancers, such as liver, pancreatic, brain, breast, colon, and some other tissues, including lymphoma and leukemia; CSCs also proved to be responsible for chemo-resistance, metastasis, and the relapse of tumors [9,10].

In the same context, the accumulated data show that CSCs have the ability to differentiate into multi-lineage, such as pericytes, endothelial cells, and cancer-associated fibroblasts, and they have the potential to reconstruct their microenvironment by differentiation or recruitment of other cells and, therefore, contribute to the initiation and progression of cancers [11,12,13].

Although the immune system and angiogenesis have been shown to have central roles in the tumorigenesis, the exact relationship between different blood cells and solid cancer stem cells is still unknown. In this respect, until recently, it was thought that the recruitment of blood and immune cells from other sites is the way that a tumor constructs its microenvironment, while the ability of cancer cells in solid tumors to give some types of hematopoietic cells have been reported recently [14,15,16].

Our lab has developed a CSC model that was converted from mouse induced pluripotent stem cells (miPSCs). The CSCs derived from miPSCs showed tumorigenicity, differentiation potential, and self-renewal ability when cultured in the conditioned medium (CM) from cancer cell lines [17]. CM mimics the cancerous niche, in which miPSCs are exposed to different growth factors and chemokines secreted from cancer cells. While using this method, we successfully generated CSC models using CM from lung, pancreas, breast, and liver cancer cell lines [12,17,18,19,20]. Furthermore, we showed that our CSC models could differentiate into vascular endothelial-like cells and cancer-associated fibroblasts, which support tumor growth in vitro and in vivo. The CSCs exhibited angiogenesis, self-renewal, and expressed different markers for CSCs associated with undifferentiated state and formed malignant tumors with metastases. In contrast, miPSCs without treatment formed typical teratomas without metastasis when transplanted into nude mice.

In this study, we investigated the potential of CSCs to give hematopoietic cells. The morphology and characteristics of non-adherent cells (NACs) that arise from adherent CSC model were analyzed while using Giemsa staining, immunofluorescence, and flow cytometry. The ability of NACs homing to the bone marrow was also assessed in vivo. Furthermore, CSCs were investigated for their ability to give rise to macrophages by inducing their differentiation with interleukin 3 (IL3) and stem cell factor (SCF). Our results showed that CSCs might be a source of different hematopoietic cells in the tumor microenvironment and they could provide a new evidence of the chimeric origin of blood cells in the tumor microenvironment.

## 2. Results

### 2.1. Cancer Stem Cells Converted from miPSCs

**Figure 1:**
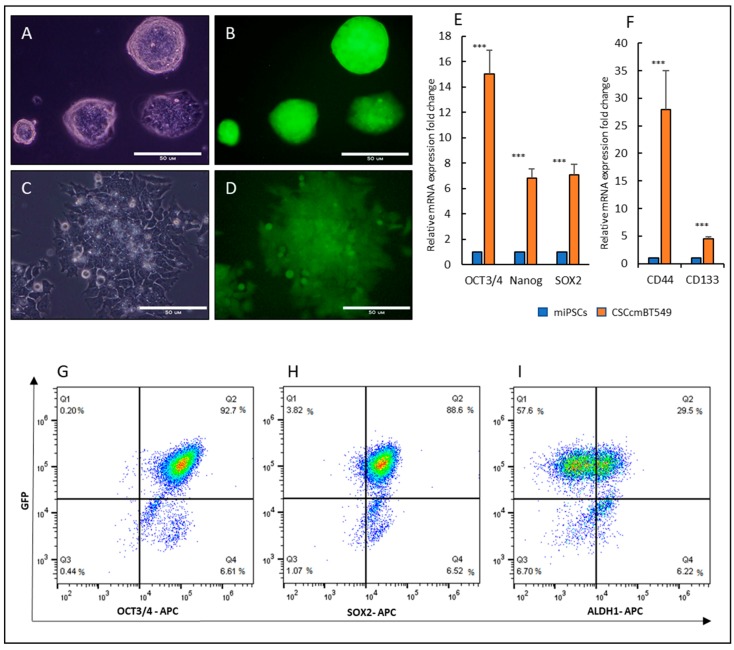
Morphology and Expression of GFP, stemness and cancer stem cell markers in CSCcmBT549 cells when compared with those in mouse induced pluripotent stem cells (miPSCs). (**A**,**B**) Representative images of miPSCs colony morphplogy using the bright field and fluorescence showing the expression of GFP in culture media with LIF. Scale bars represent 50 μm. (**C**,**D**) Representative images of CSCcmBT549 cells using bright field and fluorescence showing the expression of GFP in the cells after selection by puromycin. Scale bars represent 50 μm. (**E**,**F**) Gene expression levels of stem cell markers, OCT3/4, Nanog and Sox2 (**E**) and cancer stem cell markers, CD133 and CD44 (**F**) evaluated by RT-qPCR. The gene expression levels normalized by GAPDH expression were compared between CSCcmBT549 cells and miPSCs. (**G**–**I**) Flow cytometry analysis for stemness markers, (**G**) OCT3/4 and GFP, (**H**) SOX2 and GFP, (**I**) ALDH1 and GFP. GFP level is in parallel with Nanog expression *** *p* < 0.001.

Our lab has previously established a protocol for generating CSCs from miPSCs while using conditioned media from different cancer cell lines. The conditioned media of human breast cancer cell line BT549 cells was used to convert miPSCs into CSCs, CSCcmBT549 cells [12]. The CSCs showed tumorigenic ability in vivo and the expression of stemness markers in addition to the ability to differentiate into fibroblast like cells with the character of cancer associated fibroblasts. Here, we used the primary cultured cells, which had been isolated from the primary tumor that was developed from CSCcmBT549 cells and showed the characteristics of tumorigenicity and stemness. CSCcmBT549 cells were maintained in the presence of a 10% conditioned medium without Leukemia Inhibitory Factor (LIF), while miPSCs were cultured in the presence of LIF. In culture condition with LIF, miPSCs make colonies and show green fluorescent protein (GFP) expression (Figure 1A,B), while the CSCcmBT549 cells proliferate and survive in the absence of LIF showing GFP expression under the Nanog promoter indicate the expression of Nanog (Figure 1C,D). In contrast, miPSCs failed to survive and maintain undifferentiated state in the absence of LIF, as was well demonstrated in previous reports [12,17]. CSCcmBT549 cells maintained the expression of stem cell markers, such as OCT3/4, Nanog, and Sox2. Moreover, the expression of cancer stem cell markers, CD133 and CD44, elevated in CSCcmBT549 cells when compared with those in miPSCs (Figure 1E,F). Flow cytometry also confirmed the expression of stemness markers (Figure 1G–I).

### 2.2. Non-Adherent Round Cells Emerging from CSCs

**Figure 2:**
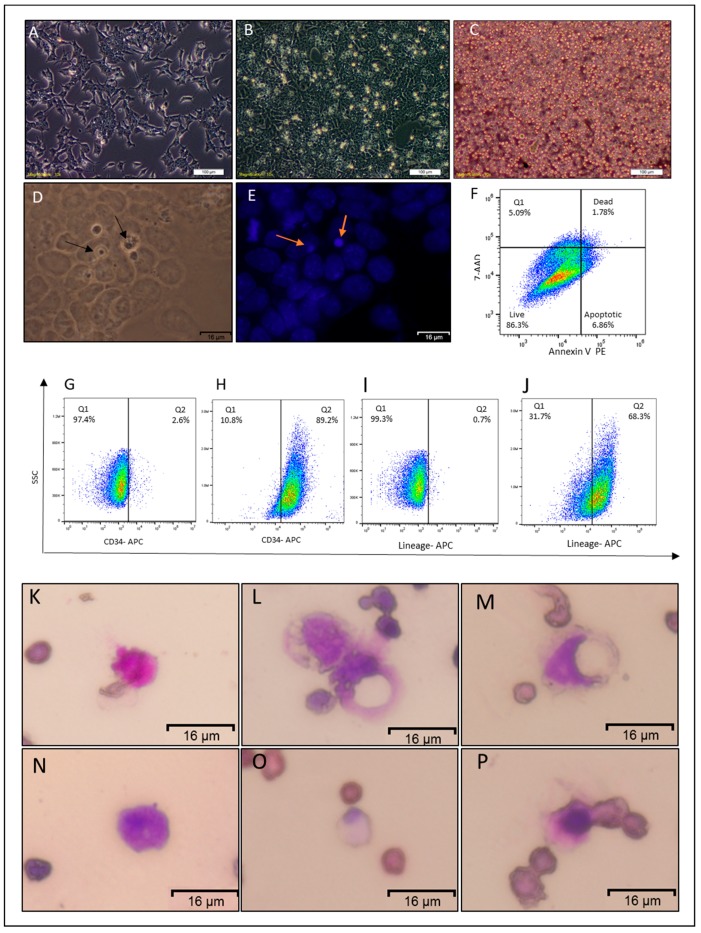
Characterization of the non-adherent round cells. (**A**) Representative image of CSCcmBT549 after 24 h of seeding. (**B**) Representative images of CSCcmBT549 cells after 72 h of seeding, showing round non-adherent cells on the top of the monolayer of adherent cells. (**C**) Floating non-adherent cells collected from the culture of CSCcmBT549 cells. Scale bars for (**A**,**B**,**C**) represent 100 μm. (**D**,**E**) Bright field and DAPI staining showing nuclei of round non-adherent cells (NACs) on the top of the monolayer adherent cells. Scale bars represent 16 μm. (**F**) Representative image of flow cytometry analysis of apoptosis assay by” Annexin V and 7-AAD kit” shows that the majority of the cells are viable while apoptotic and dead cells are less than 15%. This image is representative of at least three independent experiments. (**G**–**J**) Flow cytometry analysis for CD34 and hematopoietic lineage differentiation markers (Lineage Cell Detection Cocktail-Biotin, where (**G**,**I**) are for adherent CSCcmBT549 cells and (**H**,**J**) are for NACs. Each result is shown as a representative of at least three independent experiments. (**K**–**P**) Wright–Giemsa staining of floating cells showing different diameters and staining patterns. Scale bars represent 16 μm.

CSCcmBT549 cells have both GFP and puromycin resistance genes that are expressed under Nanog promoter, allowing for eliminating differentiated and host-derived cells from CSCs after the culturing of primary cells from mouse allografts. CSCs from the primary tumor were maintained in miPSCs media with 10% conditioned media. The cells were washed after 24 h of culturing to remove the non-adherent and dead cells. After 72 h of culturing, round floating or weak adherent like cells were observed on the top of the adherent monolayer of CSCs (Figure 2B). Fixing and staining cells with DAPI after 72 h showed that round like cells have nucleus staining positively with DAPI, and those cells were smaller than adherent cells (Figure 2D–E). In the next step, the floating cells were collected and found to have heterogeneous diameters with round morphology (Figure 2C). The viability of non-adherent cells (NACs) was analyzed by flow cytometry while using Annexin V and 7-AAD and showed that 86.5 ± 2% of floated cells were viable (Figure 2F).

### 2.3. NACs Have Hematopoietic Cells Characteristics

The NACs were analyzed by flow cytometry to examine the expression of hematopoietic lineage markers while using the Lineage Cell Detection Cocktail in addition to the CD34 antibody. The flow-cytometric analysis revealed that around 78.9 ± 15.6% of NACs were positive for lineage markers, and 89.3 ± 1.5% were positives for CD34 (Figure 2H,J), in contrast of parental adherent cells (Figure 2G,I).

**Figure 3:**
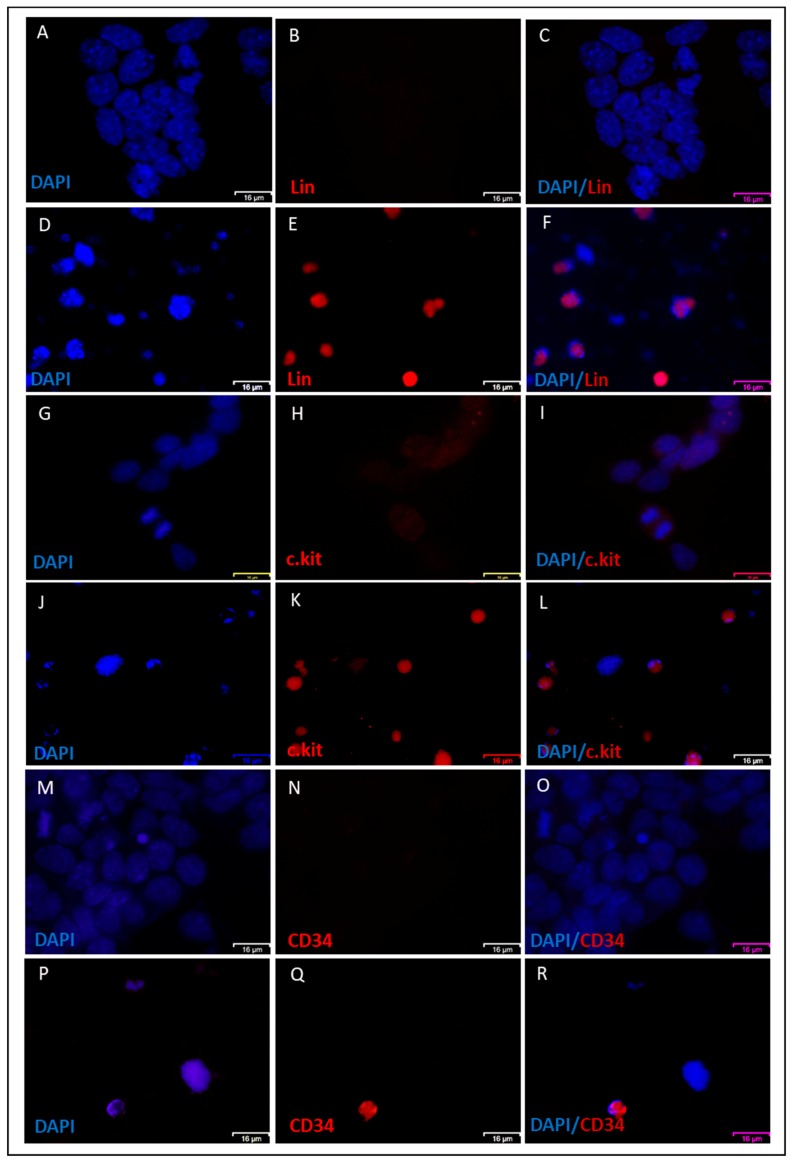
Immunofluorescence staining of NACs. (**A**–**F**) Immunofluorescence staining showing both adherent CSCcmBT549 cells (**A**–**C**) and floating cells (**D**–**F**) stained for lineage markers. (**G**–**L**) CD34 immunofluorescence staining showing both adherent CSCcmBT549 cells (**G**–I) and floating cells (**J**–**L**). (**M**–**R**) c-kit immunofluorescence staining showing both adherent CSCcmBT549 cells (**M**–**O**) and floating cells (**P**–**R**). Scale bars represent 16 μm.

**Figure 4:**
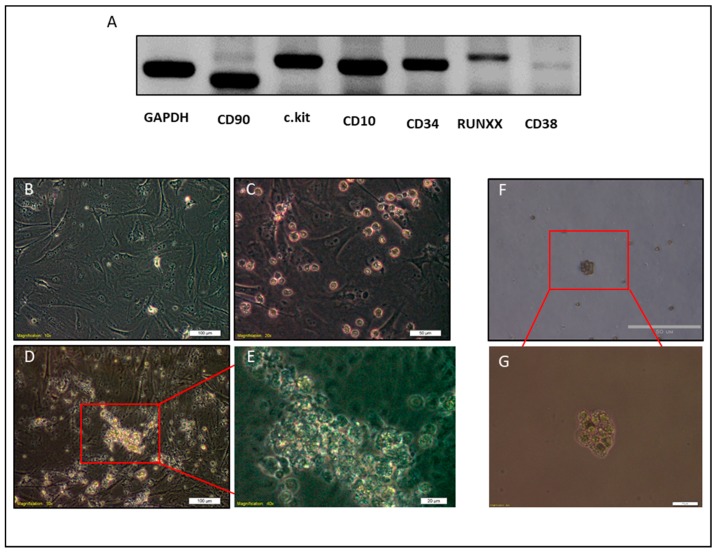
Molecular phenotyping and clonogenic test of NACs. (**A**) Agarose gel electrophoresis of NAC transcripts amplified by RT-PCR. (**B**–**E**) Representative images of clonogenic test on MEFs, (**B**) MEFs cells before seeding of NACs (**C**) MEFs and NACs cells on the day 1. (**D**,**E**) Representative images of the colony of NACs after one week of culture on MEFs. F, G) Clonogenic test on semisolid media (methylcellulose). Scale bars represent: (**B**,**D**) 100 μm, (**C**,**F**) 50 μm, and (**E**,**G**) 20 μm.

Furthermore, Wright–Giemsa staining of NACs showed heterogeneous patterns that were similar to different types of leukocytes, such as orange to pink granules in cytoplasm as eosinophils (Figure 2K), dark bluish-purple granules and reddish-purple nuclei as basophils (Figure 2N), and violet nucleus and light blue or light pink cytoplasm as monocytes (Figure 2L,M,O,P). The nuclei were also either lobed, ellipsoidal, or round (Figure 2K–P). Immunofluorescence staining also confirmed the expression of lineage markers, CD34, and c-Kit on the NAC surfaces in contrast to parental adherent cells that were negative for lineage markers and CD34 and low positive for c.kit (Figure 3A–R). Consistent with these findings, molecular phenotyping revealed that NACs expressed different hematopoietic cell markers, such as CD34, CD38, CD10, c-Kit, CD90, and RUNX1 (Figure 4A).

NACs were also cultured on mouse embryonic fibroblast (MEFs) and semisolid media, methylcellulose, to investigate their ability to form colonies. Culturing NACs on MEFs or methylcellulose showed the ability of those cells to form colonies. However, the size of colonies on MEFs was bigger than that on methylcellulose (Figure 4B–G).

### 2.4. NACs Showed the Ability of Homing to Bone Marrow and Might Lead to Restoring Leukocytes in Busulfan-Conditioned Mice

**Figure 5:**
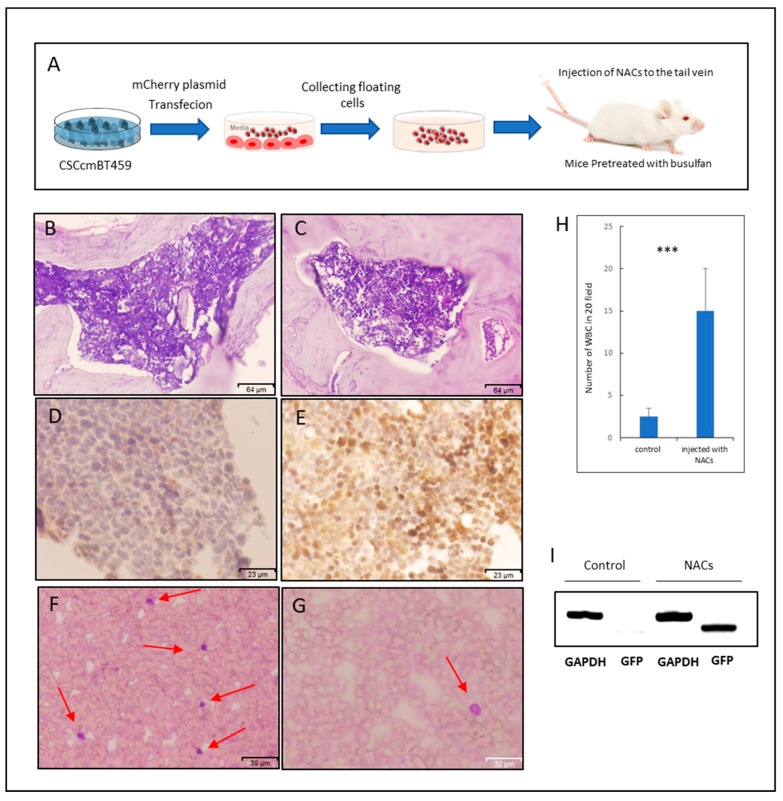
Engraftment and homing of NACs in the bone marrow. (**A**) Schematic graphic of preparation and injection of NACs. (**B**–**E**) Representative images for the sections of the bone marrow stained with Hematoxylin and Eosin (**B**,**C**) and immunostained for anti-mCherry (**D**,**E**), where (**B**,**D**) are sections of mice injected with PBS as controls and (**C**,**E**) are those from mice injected with NACs. (**F**,**G**) Representative images of peripheral blood smears stained with Wrights–Giemsa, (**F**) for mice injected with NACs and (**G**) for mice injected with PBS as controls. Red arrows indicate the WBCs. (**H**) Average number of WBCs in 20 random fields of three independent peripheral blood smears stained with Wrights-Giemsa. (**I**) Agarose gel electrophoresis of PCR products amplified from DNA samples from peripheral blood after four weeks of injection of both mice injected with PBS as controls and mice injected with NACs. *** indicate the *p* ≤ 0.001.

NACs were injected into the tail vein of Balb/c nude mice to assess the potential of clonogenicity in vivo and the engraftment of NACs. Before the injection, CSCcmBT549 cells were transfected with a plasmid expressing mCherry and stable transfectants were selected. Transfected CSCcmBT549 cells expressed both GFP and mCherry (Supplementary Figure S1A–C). However, the GFP expression was lost when the cells differentiate, since Nanog is a stemness marker. They expressed mCherry, which enabled the tracking of daughter cells derivate from CSCcmBT549 cells (Supplementary Figure S1D–F). The mice that were conditioned with busulfan were injected with two doses of NACs. Four weeks after injection, the cell suspensions obtained from the bone marrow (BM) and the spleen showed red fluorescent cells (Supplementary Figure S1J–L,P–R). The engraftment of NACs and the presence of NACs in the mouse BM were further confirmed by immunohistochemical staining with antibody against mCherry, which showed strong positive staining of BM sections when compared with those in mice that were injected with PBS as controls (Figure 5D,E). Although the mCherry positive cells were detected in BM cells after isolation while using fluorescence microscope and by immunohistochemical staining of BM sections, there was a difference regarding the number of positive cells depending on the method used to detect cells. It seemed that the number of mCherry positive cells in isolated cells that were detected by fluorescence microscope is less than those in the BM sections that were stained while using mCherry antibody (Figure 5E and Supplementary Figure S1L). Notably, the counting of 20 random fields of peripheral blood smears stained with Wright–Giemsa showed that the number of white blood cells (WBC) was significantly higher in mice that were injected with NACs than in controls injected with PBS (Figure 5F–H). Moreover, the PCR reaction confirmed the presence of cells that were derived from NACs in the peripheral blood, which have a GFP gene, after four weeks of injection of NACs in the tail vein (Figure 5I). The increasing number of WBCs and the presence of cells derived from NACs may reflect the ability of NACs to restoring and repopulating of WBCs through migration and homing.

### 2.5. Differentiation of CSCs into Macrophages

**Figure 6:**
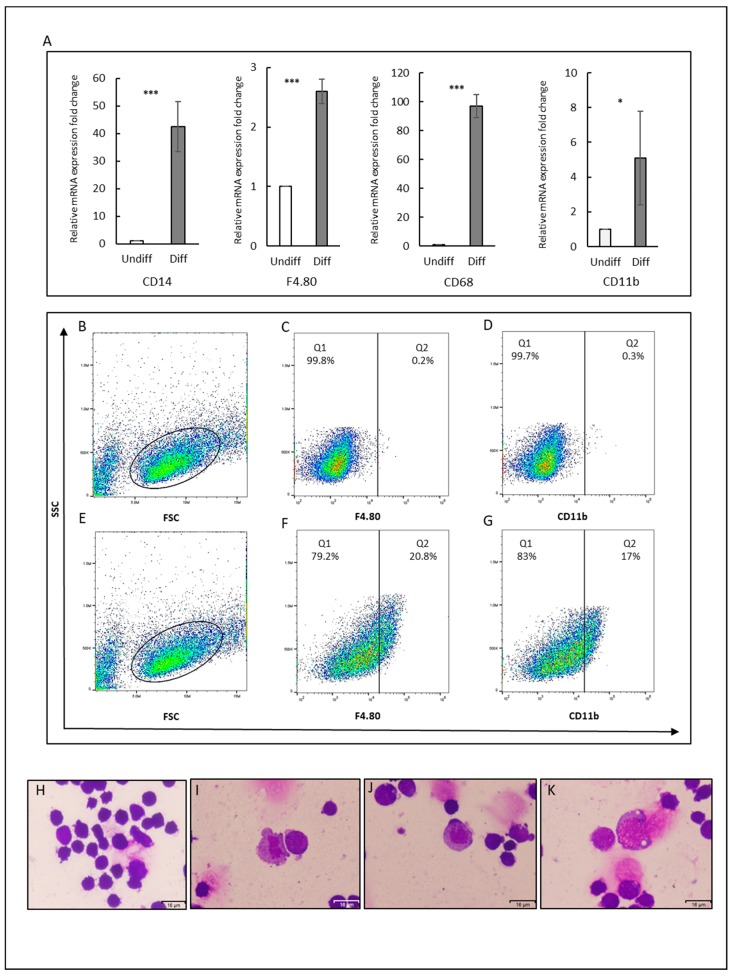
Differentiation potential of CSCs into macrophages. (**A**) Gene expression levels of macrophage markers (F4/80, CD11b, CD14, and CD68). The gene expression levels normalized by GAPDH expression levels were relatively compared between the cells with/without stimulation of differentiation by IL3 and SCF. * *p* ≤ 0.05; *** *p* ≤ 0.001. Diff: differentiated cells, Undiff: undifferentiated cells. (**B**–**G**) Flow cytometry analysis for F4.80 and CD11b. (**B**–**D**) Cells without stimulation of differentiation. (**E**–**G**) Cells with stimulation of differentiation. (**B**,**E**) FSC and SSC distribution of gated cells. (**C**,**F**) F4.80 and (**D**,**G**) CD11b. Each result is shown as a representative of at least three independent experiments. (**H**–**K**) Representative images for CSC smears stained with Wrights–Giemsa. (**H**) For control and (**I**,**G**,**K**) Cell smears after differentiation showing irregular shape with pseudopods (**I**,**K**), cytoplasmic vacuolization (k), and lobed (**I**,**K**), or Ovid nucleus (**J**). Scale bars represent 16 μm.

We applied in vitro differentiation protocol to direct differentiation of CSCs into macrophages in an attempt to evaluate the potential of adherent cancer stem cells to differentiate into specific hematopoietic cell types. The differentiation was stimulated by adding IL3 and SCF to the culture media. After 12 days of differentiation, the expression of macrophage surface markers: F4/80, CD11b, CD14, and CD68 significantly increased (Figure 6A). Interestingly, the expression of CD14, which defines the characteristic of macrophages, was approximately 40-fold higher in the differentiated cells than in the undifferentiated CSCs. CD68 was considered as a marker of tumor associated macrophages elevated approximately 100-fold. We further analyzed the subpopulation expressing F4/80 and CD11b in both undifferentiated and differentiated CSCs by flow cytometry to evaluate the overall differentiation process. The cells expressing F4/80 and CD11b after differentiation were found increasing to 15.2 ± 6.4% and 11.7 ± 4.7% respectively (Figure 6B–G).

Moreover, Wright–Giemsa staining showed that some of the CSCs exhibited the morphology of macrophages after differentiation (Figure 6I–K). The irregular shape with pseudopods, with vacuoles in cytoplasm and lobed or Ovid nucleus was observed. In contrast, the undifferentiated cells showed dark purple staining in whole cells (Figure 6H).

## 3. Discussion

The characteristics of stemness in cells reflect the ability to perpetuate their lineage and give rise to differentiated phenotypes. In the case of CSCs, cell phenotypes derived from CSCs are still under investigation [21]. There is substantial evidence in favor of the multipotentiality and the ability of CSCs to form tumor microenvironment along with other tissue components by providing different cell phenotypes, such as endothelial cells and cancer-associated fibroblasts [12,22,23,24]. Hematopoiesis includes many progenitor and cell types, which could take place in the bone marrow, the liver, or the yolk sac, depending on the development stage [25]. Responding to the changes in the architecture of tissue, paracrine and autocrine feedback loops of different cytokines and chemokines could direct CSCs to different fates. It is well established that targeting CSCs reduces relapse and metastases in cancer patients. Moreover, by combining CSCs, targeted therapies with conventional one tumors could be eradicated [26]. Targeting stemness pathways and microenvironment components while using stemness markers, or immunological approaches are some examples of recent efforts for designing more effective cancer treatments [27,28]. Those strategies have proven to be valid and they limit tumor progression, in addition to the reduction of tumor size [29,30]. New cells that arise from CSCs could drive tumor phenotype, survival, and growth [31,32]. Here, we describe that CSCs could give non-adherent cells with a phenotype common to hematopoietic cells.

Our lab has developed a unique method for obtain CSCs from iPSCs in the presence of conditioned media from cancer cell lines mimicking tumor microenvironment. After conversion, the cells that were injected into mice exhibited the ability to form malignant tumors, while iPSCs developed non-malignant teratoma. Moreover, isolated cells from malignant tumors expressed cancer stem cell markers and had ability to survive without LIF in culture media, while miPSCs failed to survive without LIF [12,17,18,19,33].

CSCcmBT549 cells that were used as the CSCs in this paper were well isolated and selected in the presence of puromycin allowing for eliminating all of the host-derived and differentiated cells. By culturing the CSCs, we observed that floating cells, NACs, are arising from adherent cells. Our data show that the NACs are viable and they do not adhere to gelatin-coated dishes unlike the parent cells. Moreover, the NACs staining pattern and morphology were found to be quite similar to those of typical white blood cells (WBCs). CSCs may have the ability to differentiate into early hematopoietic stem cells, which eventually give different progenitors, since the NACs showed different Wright–Giemsa staining patterns and expressed different markers of hematopoietic progenitors. Moreover, NACs demonstrated the ability of homing to the bone marrow and surviving up to four weeks after injection into the blood flow.

NACs could be a mixture of different hematopoietic progenitors and lineage cells, so that the identification of all progenitors, which could exist in NACs, might be difficult. This hypothesis is supported by the expression of different surface markers, such as CD10, CD34, CD38, c-kit, and Runx1 in NACs, and by positive immunoreactivity to the mixture of antibodies specific to different lineage-committed hematopoietic cells. Moreover, NACs that were able to migrate and home to the bone marrow after injection in the tail vein were confirmed by the detection of mCherry positive cells in BM sections and cultured cells that were isolated from BM. However, there was a difference in the number of positive cells between the two methods, which could be because of the difference in the sensitivity between the fluorescence microscope and the immunohistochemical staining. The low level of mCherry expression could be eliminated in the filter setting in the fluorescence microscope in contrast to immunohistochemical staining, which could amplify these signals and present it as low positive staining for the mCherry antibodies (Figure 5E). Another possible explanation of this difference could be because BM sections were prepared from femur bones, while BM cells were isolated from both femur and tibia bones. Yet, both methods detected the homing of injected NACs to the bone marrow. This is of particular interest, because NACs were shown to contain not only differentiated cells, but also hematopoietic progenitors with the potential of homing to the bone marrow and surviving. Another interesting observation was the increasing of WBCs number in peripheral blood for the mice that were injected with NACs in contrast of those in the control mice. At the same time, the WBCs had shown to have the GFP gene (Figure 5I), which confirms that there are some cells derived from NACs in the mice peripheral blood after four weeks of the injection. Thus, NACS could be responsible for the repopulation of peripheral blood cells being producing blood cells. However, more investigation could provide more explicit evidence for this association by using additional cell tracking methods.

From a perspective of cell biology, the interaction between tumor cells and other cellular components of tumor microenvironments is the main enforcement of spreading and proliferation of tumors [34]. Some recent reports showed that solid tumor progress could result in changes in peripheral blood cells, although there is a lack of studies on the ability of solid CSCs or cell lines derived from solid cancers to differentiate into hematopoietic cells. Ivana Z Matić et al. showed that there are changes in the granulocyte and lymphocyte percentages in patients with metastatic colorectal cancers [35]. While Rocca et al. showed an increase in peripheral blood natural killer cells proportions in patients with colorectal cancer [36]. However, the origin of those cells has not been investigated and is supposed to be all derived from the bone marrow.

On the other hand, the origin of tumor-associated macrophages (TAMs) now is still debated. The well-believed concept of circulating monocytes as the origin of TAMs has recently changed and embryonic-derived macrophages were considered as major suppliers to the tissue-resident macrophages [8]. Moreover, several cell lines that were derived from solid cancer and human tumor samples have been shown to be able to generate erythroid cells [16]. In the same context, polyploid giant cancer cells with stem cell-like characteristics have also been shown to have the ability to give erythroid cells [16,37,38]. Our data here also show that CSCs could be differentiated into macrophages responding to the specific factors, such as IL3 and SCF. This result could provide new insights into the origin of TAMs, as driven by cancer stem cells.

Collectively, perceptions are changing regarding the association between different types of blood cells and solid cancer cells. Within the context, the cancer stem cell model suggests the differentiation potential of CSCs into different phenotypes in the tumor microenvironment. Our data could shed the light on a new area of relationship between CSCs and hematopoietic cells in the response to tumor microenvironments. This study could encourage more investigation regarding the ability of solid cancer cells to generate more specific types of hematopoietic progenitors or differentiated cells.

## 4. Materials and Methods

### 4.1. Cell Culture

Cancer stem cells, CSCcmBT549 cells, were obtained by the conversion of miPSCs (iPS MEF-Ng-20D-17) cells in the presence of conditioned medium from human breast cancer cell line BT549 cells (ATCC HTB-122), followed by the primary culture of the tumor formed in Balb/c nude mice [12]. The stemness and tumorigenicity of CSCcmBT549 cells had previously been confirmed.

The CSCcmBT549 cells were maintained in 0.1% gelatin coated 60 mm-dishes with DMEM media (Wako, Tokyo, Japan) supplemented with 10% fetal bovine serum (FBS), 0.1 mM MEM non-essential amino acids (NEAA) (Gibco, Waltham, MA, USA), 2 mM L-glutamine (Nacalai Tesque, Kyoto, Japan), 50 U/mL penicillin/streptomycin, 0.1 mM 2-mercaptoethanol (Millipore, MA, USA), and 10% conditioned media from BT549 cells. Host derived cells were removed in the presence of 1 ug/mL puromycin (Sigma-Aldrich, St. Louis, MO, USA) in the culture medium, since CSCcmBT549 cells have both puromycin resistance and GFP gene expression under Nanog promoter. BT549 cells were cultured in RPMI-1640 medium (Wako, Tokyo, Japan) containing 10% FBS (Gibco, Waltham, MA, USA), when the cells reached 70–80% confluence, the media was changed to RPMI-1640 containing 5% FBS after 48 h to prepare the conditioned media (CM). Subsequently, CM was collected, centrifuged at 1000 g for 10 min, and the supernatant was then filtered through 0.45 μm filters (Sartorius, Göttingen, Germany). After confirming no BT549 cells, the CM was used for the maintenance of CSCs.

### 4.2. Collection and Culture of NACs

CSCcmBT549 cells, 2 × 10^6^ cells, were seeded on T75-flasks (TPP Techno, Switzerland) coated with 0.1% gelatin in the same media mentioned above. After 24 h of culture, the cells were washed with phosphate-buffered saline (PBS) and fresh media was added. At day 3, the floating non-adherent cells were harvested in 50 mL-centrifuge tubes (Corning, New York, NY, USA), centrifuged at 500g for 10 min., suspended in 0.5 mL of MyeloCult^TM^ M5300 media (stem cell technology, Vancouver, BC, Canada), and then seeded on 60-mm dishes coated with 0.1% gelatin. After 24 h, the non-adherent cells were transferred to new 60-mm dishes that were coated with 0.1% gelatin to remove CSCcmBT549 cells remained adherent.

### 4.3. RNA Extraction, cDNA Synthesis, and PCR

Total RNA was extracted from cells while using TRIzol Reagent (Thermo Fisher, Waltham, MA, USA) and then treated with DNase I (Promega, Madison, WI, USA) to remove genomic-DNA contamination from the samples. The RNA purity was evaluated by A260/A280 ratio while using NanoDrop (GE Healthcare, Chicago, IL, USA) to confirm in the range of 1.8 to 2. According to the instructions that were provided with the kit, 5 µg of RNA was reverse transcribed while using the GoScript™ Reverse Transcription System (Promega, Madison, WI, USA). The PCR reactions were performed while using the 2× Taq master mix (New England BioLabs, Ipswich, MA, USA) and the transcript products were visualized on agarose gel while using ethidium bromide (Sigma Aldrich, St. Louis, MO, USA) and 100 pb ladder (Takara, Shiga, Japan). Quantitative real-time PCR (RT-qPCR) was performed with LightCycler^®^ 480 and Light Cycler 480 SYBR Green I Master (Roche, Basel, Switzerland), according to the manufacturer’s instructions. The melting curve analysis was also undertaken to check the specificity of amplification. GAPDH was employed as a reference gene and GAPDH expression levels normalized all data. Primers were designed with bioinformatics tools, such as BLAST (NCBI, Bethesda, MD, USA) and Primer3 tool at http://bioinfo.ut.ee/primer3-0.4.0/, ensuring the primer specificity (Table 1).

### 4.4. Flow Cytometry

The NACS were washed with cold PBS, blocked with mouse FcR blocking reagent (Miltenyi Biotec, Bergisch Gladbach, Germany) for 10 min at 4 °C, and then stained with lineage cell detection cocktail-biotin antibody (Miltenyi Biotec, Bergisch Gladbach, Germany) and anti-CD34 rabbit antibody (Santa Cruz Biotechnology, Dallas, TX, USA). After incubation and washing, the cells were incubated with the secondary antibodies, Alexa Fluor 647 goat anti-rabbit IgG, and APC labeled anti-Biotin antibody (Miltenyi Biotec, Bergisch Gladbach, Germany). NACs were also stained with PE Annexin V Apoptosis detection kit, according to the manufacturer’s protocol (BdBiosciences, San Jose, CA, USA) for the detection of necrotic, apoptotic, and live cells. CSCcmBT549 cells that were stained with anti-Oct3/4 antibody (Cell Signaling Technology, Danvers, MA, USA), anti-Sox2 (Cell Signaling Technology, Danvers, MA, USA), anti-ALDH1 (Abcam, Cambridge, United Kingdom), anti-F4/80 antibody (Abcam, Cambridge, United Kingdom), and anti-CD11b (Santa Cruz Biotechnology, Dallas, TX, CA, USA). Alexa Fluor 647 Goat anti-rat IgG (Abcam, Cambridge, United Kingdom) and APC rat anti-mouse IgG (BioLegend, San Diego, CA, USA) were used as the secondary antibodies. The cells were run on the flow cytometer Accuri C6 Plus (BD Bioscience, San Jose, CA, USA) and then analyzed by Flowjo software, excluding the patterns of cell debris and aggregates based on scatter signals.

### 4.5. Wright-Giemsa Staining

The NACs were centrifuged at 800 g for 10 min., washed with PBS, and then suspended in PBS. The smears of cell suspension were prepared on slide glass that was coated with poly-L-lysine solution 0.1% (Sigma-Aldrich, St. Louis, MO, USA). The slides were left to dry, fixed with methanol, and then stained with Wright–Giemsa solution (Muto Pure Chemicals, Japan).

### 4.6. Immunofluorescence Staining

NACs smeared on slides and adherent CSCs cultured on cover slides that were coated with 0.1% gelatin were fixed while using 4% paraformaldehyde (Wako, Tokyo, Japan) for 20 min., washed two times with PBS, and then blocked using PBS containing 10% FBS at room temperature for one hour. The cells were then incubated overnight at 4 °C with primary antibodies, lineage cell detection cocktail-biotin antibody (Miltenyi Biotec, Bergisch Gladbach, Germany), anti-CD34 antibody (Santa Cruz Biotechnology, Dallas, TX, USA), and anti-CD117/c-kit (Zytomed systems, Berlin, Germany). After incubation, the cells were washed three times with PBS and then incubated with secondary antibodies, Alexa Fluor 555 labeled anti rabbit IgG goat antibody (Thermo Fisher, Waltham, MA, USA), APC labeled anti-Biotin antibody (Miltenyi Biotec, Bergisch Gladbach, Germany), and PE labeled anti-mouse IgG goat antibody (BioLegend, San Diego, CA, USA) for 1 h. Thereafter, they were washed three times with PBS and then mounted with VECTASHIELD^®^ Antifade Mounting Medium with DAPI (Vector Laboratories, Burlingame, CA, USA). The cells were observed with the Olympus FV-1000 microscope (Olympus, Tokyo, Japan).

### 4.7. Clonogenic Assay

MEFs (Reprocell, Japan) were seeded on 60-mm dishes at a density of 5 × 10^5^ cells with DMEM media containing 10% FBS to evaluate the ability of NACs to form colonies on mouse embryonic fibroblasts (MEFs). After 24 h, the MEFs were washed with PBS and fresh DMEM media containing 10% FBS, 0.1 mM MEM non-essential amino acids (NEAA), 2 mM L-glutamine, 50 U/mL penicillin/streptomycin, 0.1 mM 2-mercaptoethanol, and 10% conditioned media were added and 1 × 10^5^ NACs were seeded on the top of MEFs. One week after NACs seeding, colony formation was assessed under inverted microscope IX81 (Olympus, Tokyo, Japan). MethoCult™ GF M3434 media (Stem Cell Technology, Vancouver, BC, Canada) was employed for the clonogenic test on semisolid media. The NACs were cultured in MethoCult™ media according to the manufacturer’s protocol. Colony forming ability was evaluated two weeks after.

### 4.8. Introduction of mCherry Gene

mCherry expressing plasmid pAcmCherry-C2 was constructed by replacing AcGFP1 gene in the plasmid pAC-GFP1-C2 (Takara, Shiga, Japan) with mCherry gene. Two µg of pAcmCherry-C2 DNA was transfected to 2 × 10^6^ CSCcmBT549 cells that were suspended in 600 µL of Gene Pulser electroporation buffer (Bio-Rad Laboratories, Hercules, CA, USA). The cell suspension was electroporated in 0.4-cm gap-cuvette by Gene Pulser II (Bio-Rad Laboratories, Hercules, CA, USA) and the cells were seeded in gelatin-coated dishes. Afterwards, G418 resistant transfectants were selected by culturing for one to two weeks in the presence of G418 (Wako, Tokyo, Japan) at a concentration of 300 ug/mL. mCherry- and GFP-positive undifferentiated cells were both further selected in the presence of 1 ug/mL puromycin (Sigma-Aldrich, St. Louis, MO, USA) for seven days. The media were changed daily.

### 4.9. Animal Experiments

Female Balb/c nude mice were purchased from (Charles River Laboratories, Wilmington, MA, USA) and then kept under pathogen-free conditions. All of the experiments were conducted according to the animal care and use committee of Okayama University under the project license OKU-2016252. Mice received busulfan (90 mg/kg) (LKT Laboratories, St. Paul, MN, USA) by intraperitoneal injection of 30 mg/kg/day for three days. On day 4, the mice were divided into two groups. A total of 1 × 10^7^ NACs expressing mCherry were washed with sterile PBS, suspended in 100 uL of PBS, and then injected into the tail vein of one group, whereas PBS was injected into the tail vein of the other group of mice as a control. After one week, another 5 × 10^6^ NACs were injected to increase the chance of homing.

Four weeks after injection, the bone marrow from femur and tibia bones, the peripheral blood, and spleens were obtained. The smears of peripheral blood were stained with Wright–Giemsa as described above. The BM samples were treated with red blood cell (RBC) lysis buffer (BioLegend, San Diego, CA, USA), washed with PBS, suspended in 0.5 mL of DMEM with 15% FBS, and then seeded in six-well plate while using DMEM supplemented with 15% FBS, 0.1 mM MEM NEAA, 2 mM L-glutamine, and 0.1 mM 2-mercaptoethanol. The spleen samples were chopped and suspended in dissociation buffer, as described previously [17]. Cells were observed under a fluorescence inverted microscope IX81 (Olympus, Tokyo, Japan).

### 4.10. DNA Extraction

Four weeks after injection, peripheral blood samples were obtained from mice, DNA was extracted while using blood and cell culture DNA mini kit (Qiagen, Hilden, Germany) according to the kit instructions, and the PCR reactions were performed as the same mentioned above with GAPDH and GFP primers. Primer sequences are shown in (Table 1).

### 4.11. Histological Analysis and Immunohistochemistry

Extracted femur bones were decalcified while using 14% EDTA (Wako, Tokyo, Japan), embedded into paraffin using standard histologic techniques and sectioned at 5 µm of thickness. The sections were deparaffinized, rehydrated and stained with hematoxylin-eosin (Hematoxylin solution, Sigma-Aldrich, St. Louis, MO, USA; 0.5% Eosin Y, Wako, Tokyo, Japan) for histological analysis. For the immunohistochemistry of mCherry, the antigen retrieval was carried out by using sodium citrate buffer (pH 6.0) with 0.05% Tween 20 for 15 min by a standard microwave heating technique. After cooling down, hydrogen peroxide blocking and Ig blocking were undertaken while using 3% hydrogen peroxide and Ig blocking reagents (Vector Laboratories, Burlingam, CA, USA). After overnight incubation of sections with anti-mCherry, rabbit (Funakoshi, Tokyo, Japan) at 4 °C, the ABC staining kit and DAB (3,3′-diaminobenzidine) substrates (Vector Laboratories, Burlingame, CA, USA) were used for the detection of mCherry, respectively. The sections were counter-stained using hematoxylin and mounted with Micromount (Leica Camera AG, Wetzlar, Germany). The staining was evaluated under light microscopy (FSX100, Olympus, Tokyo, Japan).

### 4.12. Differentiation of CSCs into Macrophages

CSCcmBT549 cells were maintained, as described above. Differentiation was performed on a gelatin-coated dishes in DMEM containing 10% FBS, 0.1 mM MEM NEAA, 2 mM L-glutamine, 50 U/ml penicillin and streptomycin, 0.1 mM 2-mercaptoethanol, 10% conditioned media, 30 ng/mL murine stem cell factor (SCF) (PeproTech, Rocky Hill, NJ, USA), and 10 ng/mL murine interleukin-3 (IL3) (PeproTech, Rocky Hill, NJ, USA). The cells were passaged when it reached approximately 80% confluence. After 12 days, cells were analyzed for macrophage markers.

### 4.13. Statistical Analysis

Statistical analyses were performed while using the Prism Software version7 (Graph Pad Software, San Diego, CA, USA). The data are presented as mean ± SD. Statistical comparisons between experimental groups were analyzed by a T-test and *p* < 0.05 considered as statistically significant.

## 5. Conclusions

Our data demonstrate that CSCs converted from miPSCs can generate cells with hematopoietic characteristics and might have the ability to repopulate peripheral blood by migration and homing to the bone marrow after injection to the blood. CSCs derived hematopoietic cells could also contribute to the tumor microenvironment; therefore, further studies on the mechanisms by which CSCs give NACs and how those cells act together with other microenvironment components may shed light on new mechanisms of the plasticity of CSCs and their adaption to the tumor microenvironment.

## Figures and Tables

**Table 1:** Sequences of primers used in the study.

Gene	Accession Number	Forward Primer	Reverse Primer
GAPDH	NM_008084	AACGGCACAGTCAAGGCCGA	ACCCTTTTGGCTCCACCCTT
Nanog	NM_028016.3	AGGGTCTGCTACTGAGATGCTCTG	CAACCACTGGTTTTTCTGCCACCG
OCT3/4	NM_013633.3	TCTTTCCACCAGGCCCCCGGCTC	TGCGGGCGGACATGGGGAGATCC
SOX2	NM_011443.4	TAGAGCTAGACTCCGGGCGATGA	TTGCCTTAAACAAGACCACGAAA
CD133	NM_001163578.1	CCTTGTGGTTCTTACGTTTGTTG	CGTTGACGACATTCTCAAGCTG
CD44	NM_009851.2	AGAAAAATGGCCGCTACAGTATC	TGCATGTTTCAAAACCCTTGC
CD90	NM_009382.3	TGCAGCTAGGGGAGTCCAGAAT	TCCAGGCGAAGGTTTTGGTT
c.kit	NM_021099.3	CGGACAGCACCAAGCACATTTACTC	AACCATCACAGAAGCCAGAAGGACG
CD34	NM_001111059.1	TGCTGCATCTAAATAACTTGAC	AGGGATCCCAGAGGTAACTG
RUNX1	NM_001111021.2	CTGCCCATCGCTTTCAAGGTG	CTATGGTAGGTGGCAACTTGTGG
CD38	NM_007646.5	TGAGAGATCAGAACTGCCAGG	GTGTCCTCCAGGGTGAACAT
CD10	NM_008604.4	GCTAGAAGTCATTTTGAAAGATGTCCT	AGTGCCATATGTTTGATCCCAGT
CD11b	NM_001082960.1	TACGTAATTGGGGTGGGAA	GTGCCCTCAATTGCAAAGAT
CD14	NM_009841.3	CTCTGTCCTTAAAGCGGCTTAC	GTTGCGGAGGTTCAAGATGTT
F4/80	NM_010130.4	CACCGGTATAGACAAGACTGACA	TCTCACCATCAGGAAGAGCA
CD68	NM_009853.1	ACTTCGGGCCATGTTTCTCT	GCTGGTAGGTTGATTGTCGT
GFP		GACAAGCAGAAGAACGGCATCAAGG	CTCAGGTAGTGGTTGTCGGGCAG

## Supplementary Materials

The following are available online at https://www.mdpi.com/2072-6694/12/1/82/s1, Figure S1: Stable transfection of CSCcmBT549 with mCherry expression plasmid and positive mCherry isolated cells from mice.
