# Supplementary material for: Hematopoietic Cells Derived from Cancer Stem Cells Generated from Mouse Induced Pluripotent Stem Cells
Source: Cancers (Basel). 2019 Dec 29;12(1):82. doi: 10.3390/cancers12010082 (PMC7016803; doi:10.3390/cancers12010082)
Supplement: Supplementary file 1 [file cancers-12-00082-s001.pdf]

cancers

MDPI

Article

Hematopoietic Cells Derived from Cancer Stem Cells Generated from Mouse Induced Pluripotent Stem Cells

Ghmkin Hassan [1,2,*], Said M. Afify [1,3], Neha Nair [4], Kazuki Kumon [4], Amira Osman [1,5], Juan Du [4], Hager Mansour [4], Hagar A Abu Quora [1], Hend M Nawara [4], Ayano Satoh [1], Maram H. Zahra [1], Nobuhiro Okada [1], Akimasa Seno [1,6], and Masaharu Seno [1,6,*]

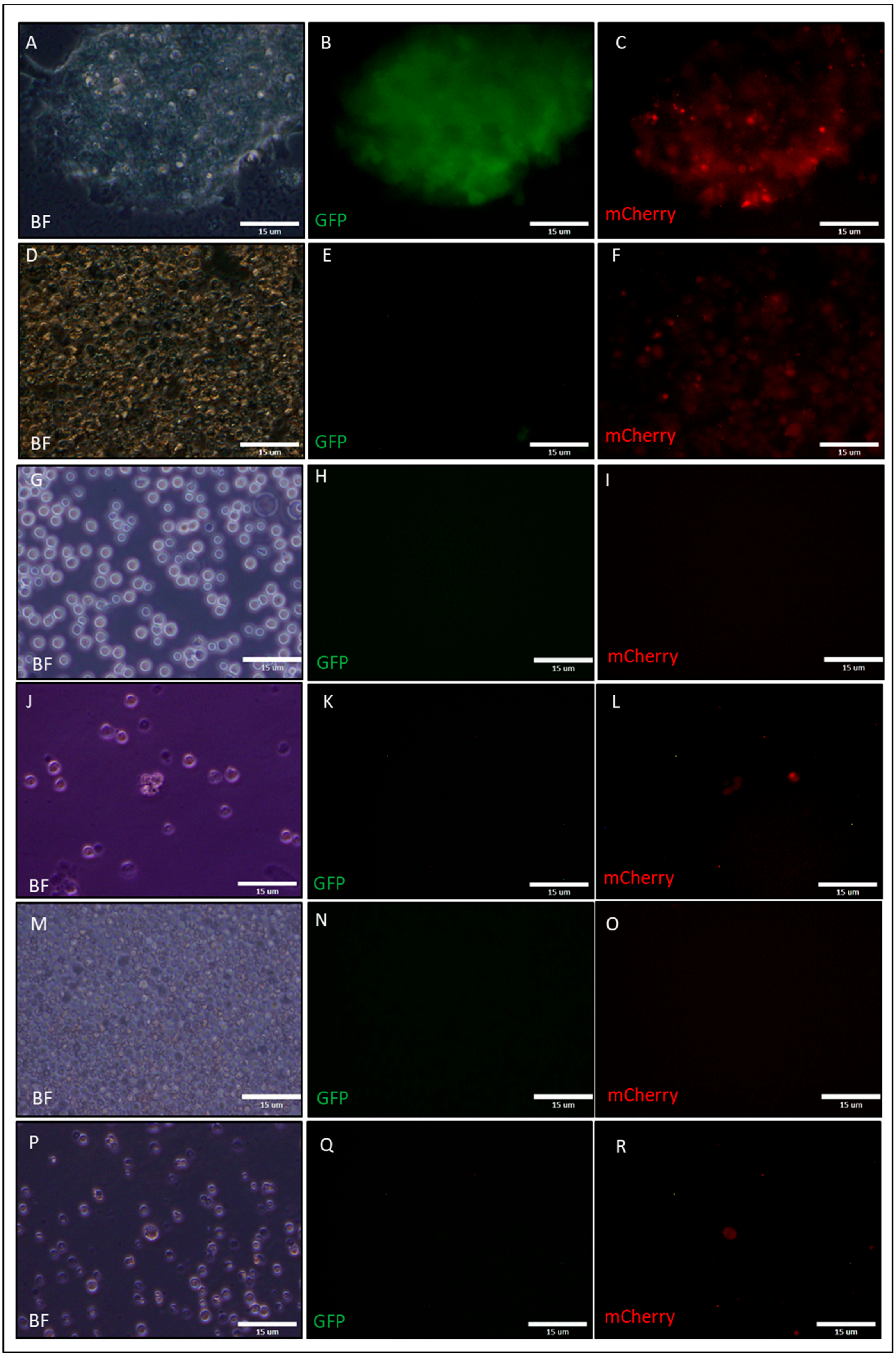

Supplementary Figure S1. Stable transfection of CSCcmBT549 cells with mCherry expression plasmid and mCherry positive cells isolated from mice. (**A**–**C**) Adherent CSCcmBT549 cells transfected with mCherry expression plasmid where (**A**) bright field, (**B**) GFP and (**C**) mCherry. (**D**–**F**) Non-adherent cells (NACs) arising from CSCcmBT549 cells transfected with mCherry plasmid, NACs

are negative for GFP and positive for mCherry where (**D**) bright field, (**E**) GFP and (**F**) mCherry. (**G–I**) Representative images of cells isolated from the bone marrow of control mice injected with PBS. (**J–L**) Representative images of cells isolated from the bone marrow of mice injected with NACs after 4 weeks of injection. (**M–O**) Representative images of cells isolated from the spleen of control mice injected with PBS. (**P–R**) Representative images of cells isolated from the spleen of mice injected with NACs after 4 weeks of injection(**J–L**, **P–R)** Show the NACs, which homed and survived in the bone marrow and the spleen. These cells are positive for mCherry but negative for GFP.
